# Morphometry of the coronary ostia and the structure of coronary arteries in the shorthair domestic cat

**DOI:** 10.1371/journal.pone.0186177

**Published:** 2017-10-11

**Authors:** Karolina Barszcz, Marta Kupczyńska, Michał Polguj, Joanna Klećkowska-Nawrot, Maciej Janeczek, Karolina Goździewska-Harłajczuk, Małgorzata Dzierzęcka, Paweł Janczyk

**Affiliations:** 1 Department of Morphological Sciences, Faculty of Veterinary Medicine, Warsaw University of Life Sciences, 159 Nowoursynowska, Warsaw, Poland; 2 Department of Angiology, Interfaculty Chair of Anatomy and Histology, Medical University of Łódź, Narutowicza 60, Łódź, Poland; 3 Department of Animal Physiology and Biostructure, Faculty of Veterinary Medicine, Wroclaw University of Environmental and Life Sciences, Kozuchowska 1/3, Wroclaw, Poland; 4 Institute of Veterinary Anatomy, Faculty of Veterinary Medicine, Freie Universität Berlin, Koserstrasse 20, Berlin, Germany; Vanderbilt University Medical Center, UNITED STATES

## Abstract

The aim of this study was to measure the area of the coronary ostia, assess their localization in the coronary sinuses and to determine the morphology of the stem of the left and right coronary arteries in the domestic shorthair cat. The study was conducted on 100 hearts of domestic shorthair cats of both sexes, aged 2–18 years, with an average body weight of 4.05 kg. A morphometric analysis of the coronary ostia was carried out on 52 hearts. The remaining 48 hearts were injected with a casting material in order to carry out a morphological assessment of the left and right coronary arteries. In all the studied animals, the surface of the left coronary artery ostium was larger than the surface of the right coronary artery ostium. There were four types of the left main coronary artery: type I (23 animals, 49%)–double-branched left main stem (giving off the left circumflex branch and the interventricular paraconal branch, which in turn gave off the septal branch), type II (12 animals, 26%)–double-branched left main stem (giving off the left circumflex branch and the interventricular paraconal branch without the septal branch), type III (11 animals, 23%)–triple-branched left main stem (giving off the left circumflex branch, interventricular branch and the septal branch, type IV (1 animal, 2%)–double-branched left main stem (giving off the interventricular paraconal branch and the left circumflex branch, which in turn gave off the septal branch). The left coronary artery ostium is greater than the right one. There is considerable diversity in the branches of proximal segment of the left coronary artery, while the right coronary artery is more conservative. These results can be useful in defining the optimal strategies in the endovascular procedures involving the coronary arteries or the aortic valve in the domestic shorthair cat.

## Introduction

In most mammals the heart is supplied with blood via the left and right coronary arteries. The coronary ostia are located in the coronary sinuses of the aortic root. According to the Nomina Anatomica Veterinaria [1], the left main coronary artery is divided into two vessels: the interventricular paraconal branch and the left circumflex branch. This is confirmed in the studies of Habermehl [2], Atalar et al. [3], Smodlaka et al. [4], Yuan et al. [5] and Kupczyńska et al. [6] in selected species of mammals. However, some authors describe a tripartite branching of the left main coronary artery. The main distal branches are the interventricular paraconal branch, the left circumlfex branch and the septal branch [7–9]. The right coronary artery is a less developed vessel, and its stem becomes the right circumflex branch.

The presence of a single coronary artery (SCA) has been described in humans. It is considered to be a rare congenital defect and often co-occurs with other congenital heart defects. 0.3–0.6% of humans are affected by abnormalities of one or both coronary ostia [10]. In veterinary medicine, a similar morphological alteration was found only in the long-tailed chinchilla (*Chinchilla lanigera*), where the right coronary artery is usually missing and only the single coronary artery exists [11].

The occurrence of additional coronary arteries has also been reported. Studies carried out on the hearts of African green monkeys (*Cercopithecus aethiops*) and crab-eating macaque (*Macaca fascicularis*) revealed the presence of a third coronary artery–TCA [12].

The assessment of the morphology of the aorta and the coronary arteries is carried out in humans not only to diagnose certain diseases, but also prior to open and endovascular cardiovascular procedures. In veterinary medicine, anatomical studies of the aorta and the coronary arteries are carried out in animals which are used as experimental models before human clinical trials [13–15]. In particular, little attention has been paid to the domestic shorthair cat heart vascularity including the coronary ostia. There is morphological study that refer only to anatomical variations of location of the coronary ostia and presence of accessory ostia either for left, right or both coronary arteries [16]. In contrast to previously publications our study presented in this paper is the first report on morphometry of coronary ostia and morphology of the stem of the left and right coronary arteries in the domestic shorthair cat. Current methods used by veterinarians for the diagnosis and treatment of coronary diseases, such as angiography, Doppler ultrasonography, magnetic resonance angiography and computed tomography or digital subtraction angiography, and especially coronary angiography require detailed knowledge of the morphometry of the coronary ostia, including clinical aspects of the subepicardial vessels [9, 17]. Such knowledge is especially useful in the explanation pathophysiology of coronary diseases [18].

The aim of this study was to measure the area of the coronary ostia, assess their localization in the coronary sinuses of the aortic root and to determine the morphology of the stem of the left and right coronary arteries in the domestic shorthair cat.

## Materials and methods

The study was carried out on 100 domestic shorthair adult feline cadavers of both genders (46 ♂ and 54 ♀), with a mean body weight of 4.05 kg, from 2 to 18 years old. The specimens were divided into two groups. The first study, which included a morphometric analysis of the coronary ostia, was carried out on samples from 52 cadavers. Hearts from the remaining 48 cadavers were injected with casting material in order to carry out a morphological assessment of the stem of the left and right coronary arteries. The animals were assigned randomly into two groups.

All the animals included in the study were euthanized by veterinary doctors at the Small Animal Clinic of the Department of Clinical Sciences, Faculty of Veterinary Medicine, of the Warsaw University of Life Sciences. The animals were not killed for the purpose of this study. The animals presented to the clinic were euthanized under general anesthesia by overdose of barbiturates with the owner’s consent for various non-cardiac reasons. The anaesthetic was administered via a peripheral intravenous catheter. The owners of the animals also consented to the use of the cadavers for scientific purposes. According to the Polish law, the *post mortem* use of tissues does not require an approval from the Ethics Committee [19]. Pathological examination of the whole body was performed before the dissection of the hearts. Nomenclature from the Nomina Anatomica Veterinaria (2012) was used [1]. The morphologic and morphometric assessment was carried out using ECLERIS (HALOLUX 150) and GLOBAL (MW 725F-I) surgical microscopes. Both devices had integrated video channels and the software for image analysis (AxioVision Rel. 4.7, Carl Zeiss MicroImaging GmbH, Jena, Germany).

### Morphometric studies of the coronary ostia

After being harvested, the hearts were washed under running-water and placed in a hypertonic NaCl solution to remove blood. Then, the pericardial sac was removed, and the ascending aorta was cut above the aortic valve commissures. Afterwards, a longitudinal cut between the aortic valve leaflets was made to visualize both coronary ostia.

Prior to the measurement, the samples were dried using filter-paper and suction. The following morphometric measurements were taken: PACS−the area of the left coronary ostium (mm^2^), PACD−the area of the right coronary ostium (mm^2^). The difference between P_ACS_ and P_ACD_ (r, mm^2^) was calculated. A statistical analysis was carried out using the STATISTICA 12 software (Dell Statistica, Tulsa, USA). The gender differences for different parameters were calculated using the NIR Fisher test and the significance level was set at p≤0.05. The results are presented in Table 1.

**Table 1:** The gender differences for P_ACS_−the area of the left coronary ostium (mm^2^), P_ACD_−the area of the right coronary ostium (mm^2^) and r–difference between P_ACS_ and P_ACD_ (mm^2^).

Gender	n	P_ACS_ (mm^2^)	P_ACS_ (mm^2^) SD	P_ACS_ (mm^2^) Min	P_ACS_ (mm^2^) Max	P_ACS_ (mm^2^) Q25	P_ACS_ (mm^2^) Median	P_ACS_ (mm^2^) Q75
		AVG						
Males	26	1,15	0,44	0,54	2,64	0,83	1,05	1,36
Females	26	1,13	0,42	0,56	2,50	0,79	1,09	1,35
Total	52	1,14	0,43	0,54	2,64	0,82	1,08	1,35
Gender	n	P_ACD_ (mm^2^)	P_ACD_ (mm^2^)	P_ACD_ (mm^2^)	P_ACD_ (mm^2^)	P_ACD_ (mm^2^)	P_ACD_ (mm^2^)	P_ACD_ (mm^2^)
		AVG	SD	Min	Max	Q25	Median	Q75
Males	26	0,51	0,34	0,12	1,30	0,28	0,35	0,72
Females	26	0,47	0,29	0,18	1,37	0,25	0,38	0,63
Total	52	0,49	0,31	0,12	1,37	0,27	0,38	0,63
Gender	n	r (mm^2^)	r (mm^2^)	r (mm^2^)	r (mm^2^)	r (mm^2^)	r (mm^2^)	r (mm^2^)
		AVG	SD	Min	Max	Q25	Median	Q75
Males	26	0,64	0,36	0,08	1,34	0,34	0,64	0,86
Females	26	0,66	0,41	0,01	1,70	0,35	0,58	0,85
Total	52	0,65	0,38	0,01	1,70	0,35	0,63	0,86

P_ACS_−the area of the left coronary ostium (mm^2^), P_ACD_−the area of the right coronary ostium (mm^2^) and r–difference between P_ACS_ and P_ACD_ (mm^2^)

### The morphologic assessment of the left main stem coronary artery

Coronary arteries were visualized by filling them with casting. Latex (LBS 3060) (Synthos Dwory Sp. z o.o, Poland) and an acrylic derivative (DURACRYL® PLUS) with additional dye (INCHEM) were injected via aorta. The hearts (n = 33) filled with the latex injection mass were fixed in a 4% formaldehyde solution. Next, the proximal segments of the left and right coronary arteries were dissected. Corrosion castings were prepared by filling selected hearts (n = 15) with acrylic. The specimens were then left at room temperature (>18°C) until the casts hardened.

After hardening, the specimens were placed in 40% KOH solution at 50°C for approximately 24 h to dissolve the organic tissue. The remnants of the dissolved tissue were removed from the specimen by continuous flushing with water for 38 h. The specimen was cleaned by a fast wash with warm water and a small amount of standard washing liquid, followed by a final flush with distilled water. The cast was later dried using airflow at room temperature for two days.

The methods of injection were successfully used as in our previous studies [6, 20–23].

## Results

**Fig 1:**
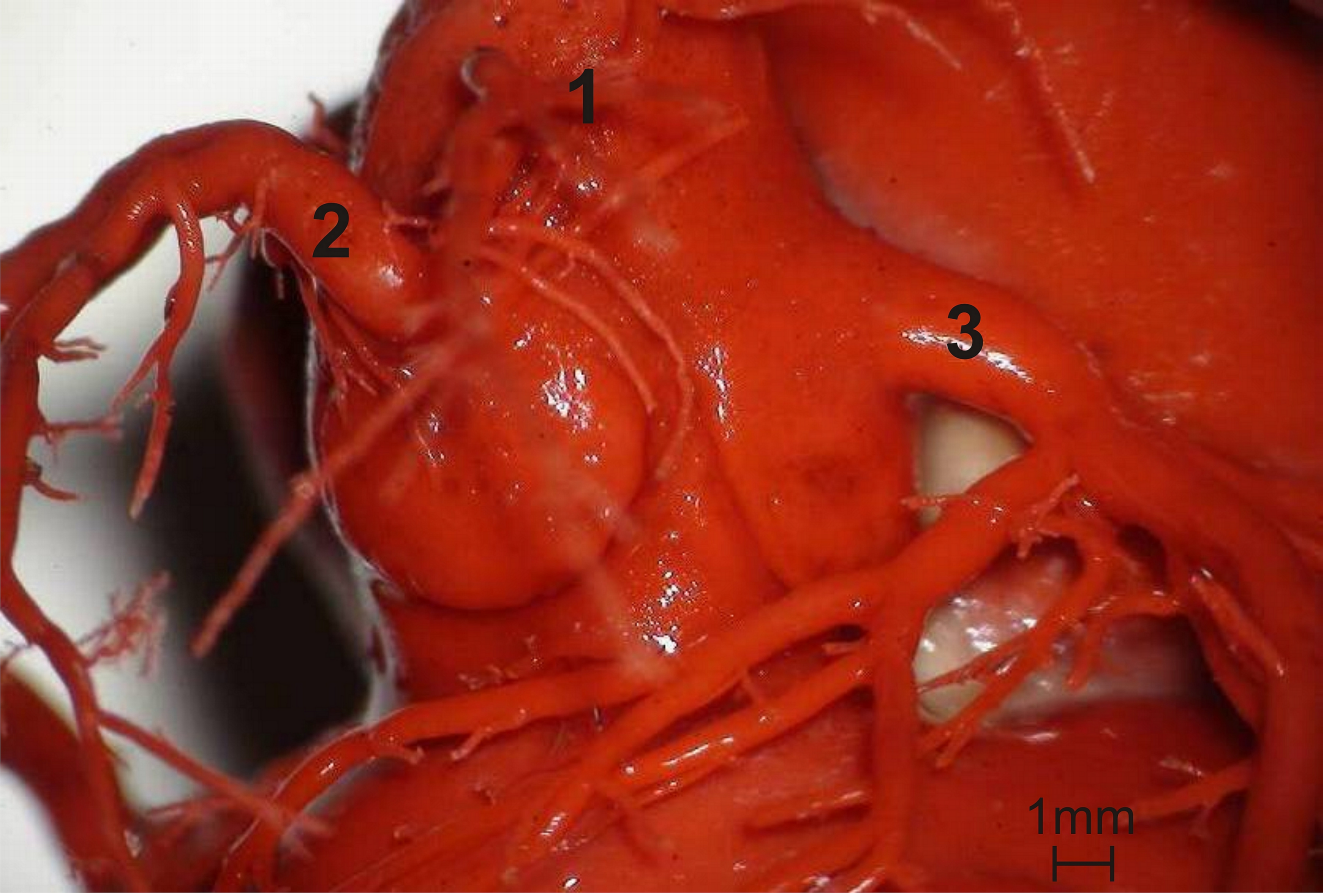
Overview of the aortic valve and ascending aorta. 1 –ascending aorta, 2 –right coronary artery, 3 –left coronary artery.

**Fig 2:**
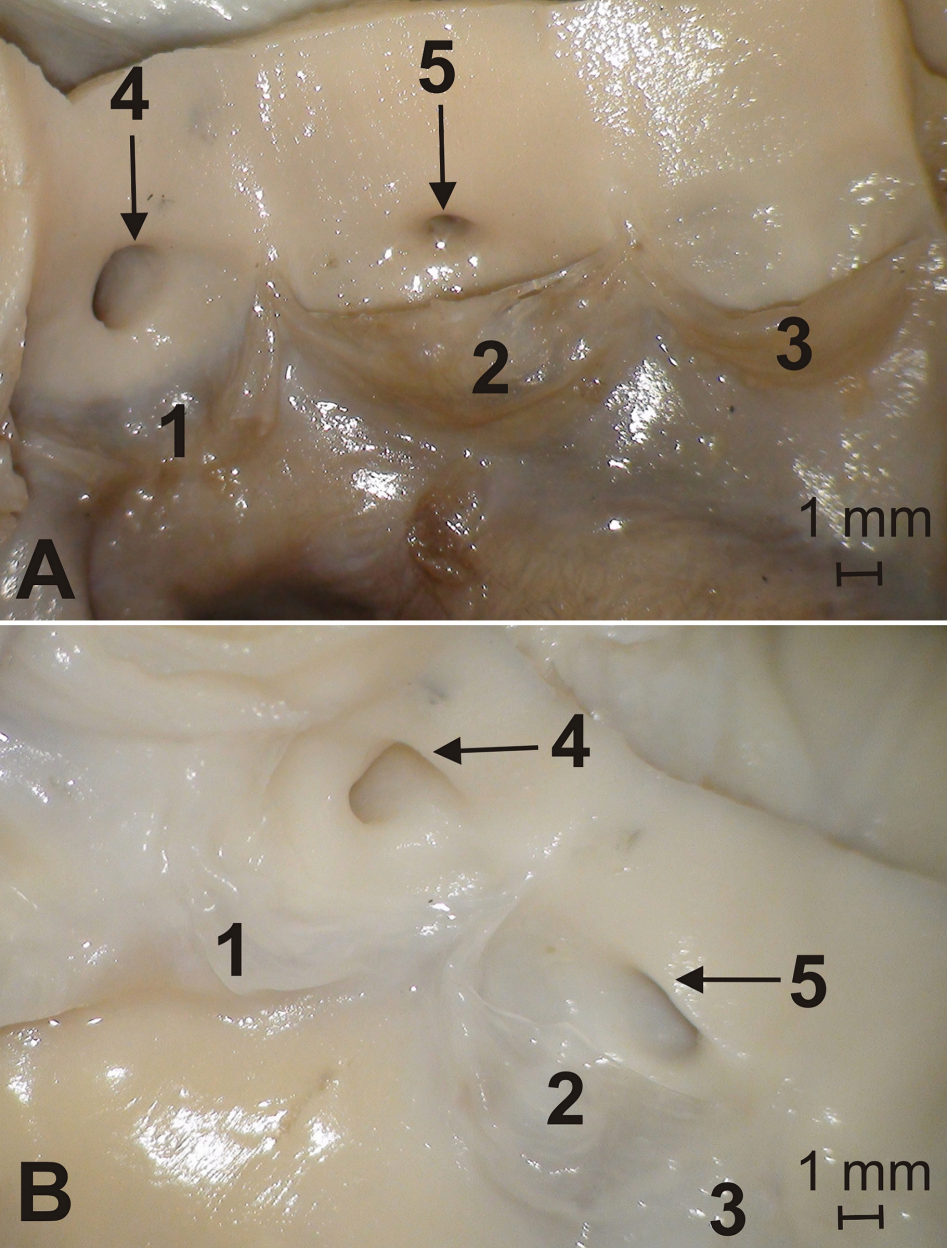
Overview of the aortic valve. 1 –left semilunar leaflet, 2 –right semilunar leaflet, 3 –non-coronary semilunar leaflet, 4 –left coronary ostium, 5 –right coronary ostium.

In all the 52 animals that underwent morphometric analyses, the aortic valve had three semilunar leaflets: the right, left and non-coronary leaflets. The right and left coronary ostia were located in the corresponding aortic valve sinuses (Fig 1). The statistical analysis of the area of coronary ostia using the NIR test did not reveal significant differences between males and females. In all the specimens, the P_ACS_ (0.54–2.64 mm^2^) was larger than the P_ACD_ (0.12–1.37 mm^2^) (Table 1). The mean difference between the area of the left and right coronary ostium was 0.65 mm^2^ (range: 0.01 mm2–1.70 mm^2^) (Fig 2). There were no statistically significant differences between males and females with regards to this parameter S1 Table.

In 47 of the 48 studied cats (98%), the left coronary artery arose from the coronary ostium located in the corresponding coronary sinus. The left main stem ran between the left auricle and the proximal part of the pulmonary trunk. Above the coronary sulcus, it divided into the left circumflex branch and the interventricular paraconal branch. The left circumflex branch entered the coronary sulcus, whereas the interventricular paraconal branch entered the interventricular paraconal sulcus. The septal branch varied the most as it was a branch of the left main stem, the left circumflex branch and the interventricular paraconal branch.

**Fig 3:**
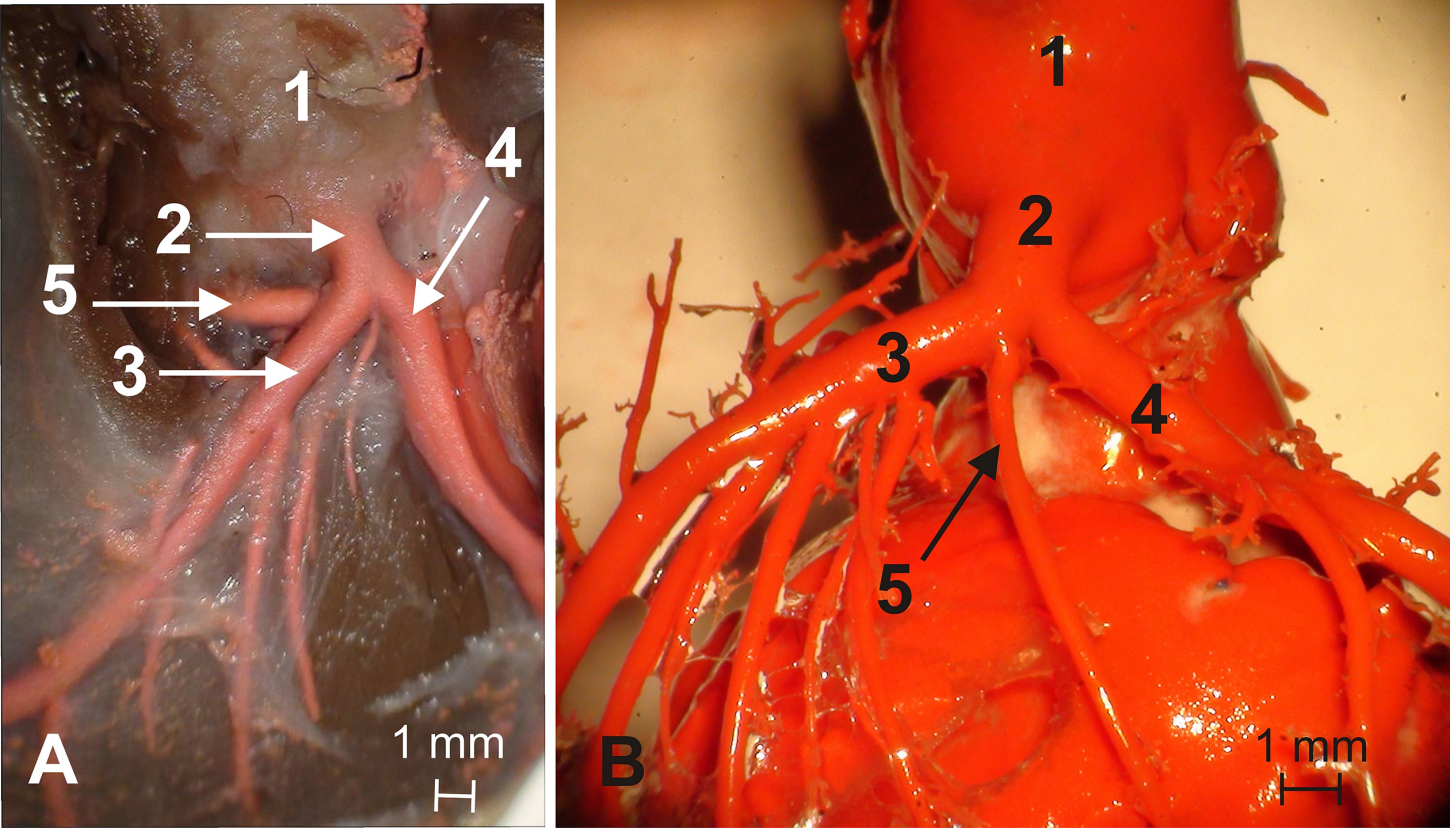
Type I–double-branched left main stem giving off the left circumflex branch and the interventricular paraconal branch, which gave off the septal branch. 1 –ascending aorta, 2 –left coronary artery (main stem), 3 –interventricular paraconal branch, 4 –left circumflex branch, 5 –septal branch.

Based on morphological differences, four types of the proximal segment of the left coronary artery were distinguished:

Type I (23 animals, 49%)–double-branched left main stem giving off the left circumflex branch and the interventricular paraconal branch, which gave off the septal branch (Fig 3)

**Fig 4:**
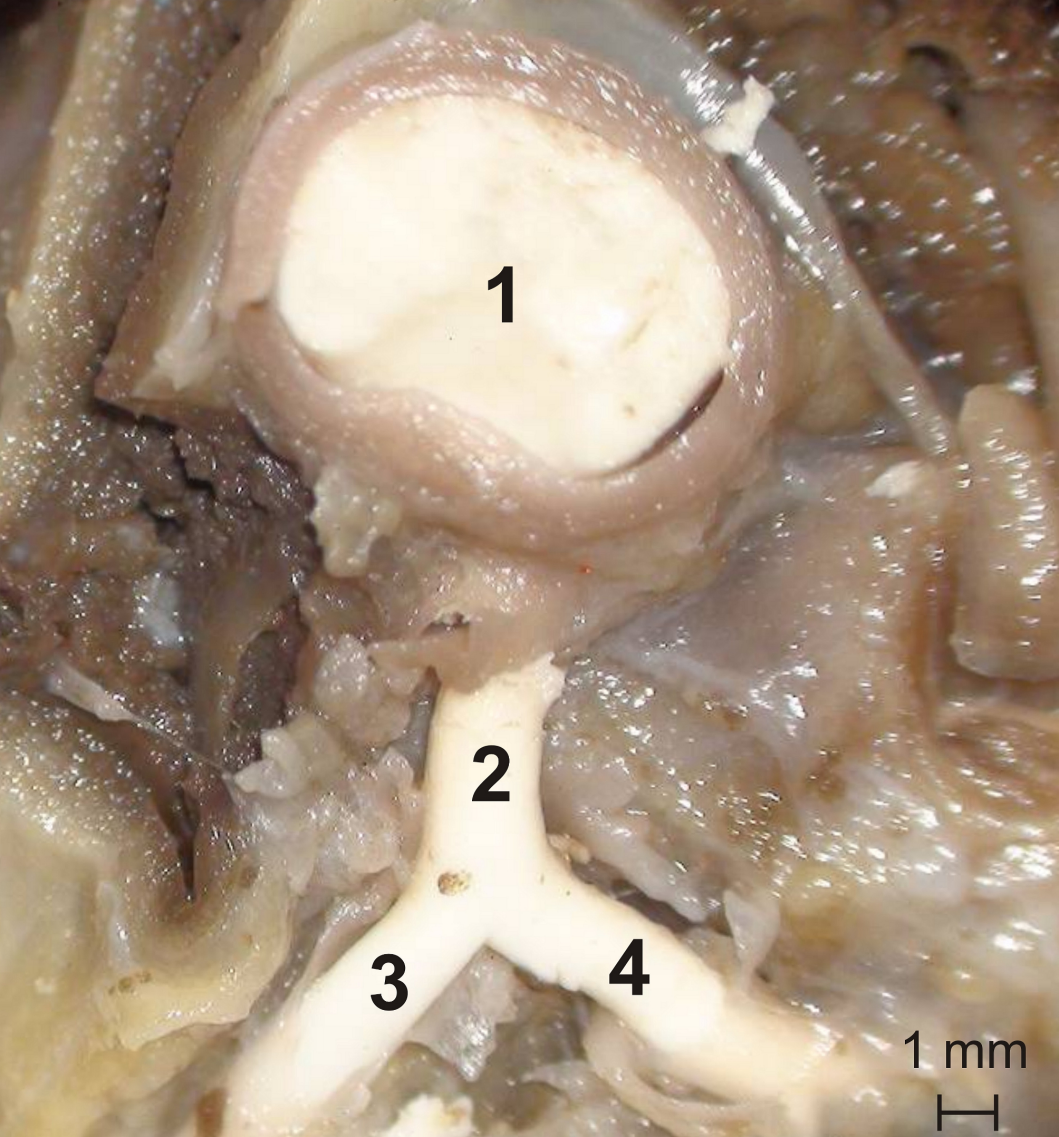
Type II–double-branched left main stem giving off the left circumflex branch and the interventricular paraconal branch without the septal branch. 1 –ascending aorta, 2 –left coronary artery (main stem), 3 –interventricular paraconal branch, 4 –left circumflex branch.

Type II (12 animals, 26%)–double-branched left main stem giving off the left circumflex branch and the interventricular paraconal branch without the septal branch (Fig 4)

**Fig 5:**
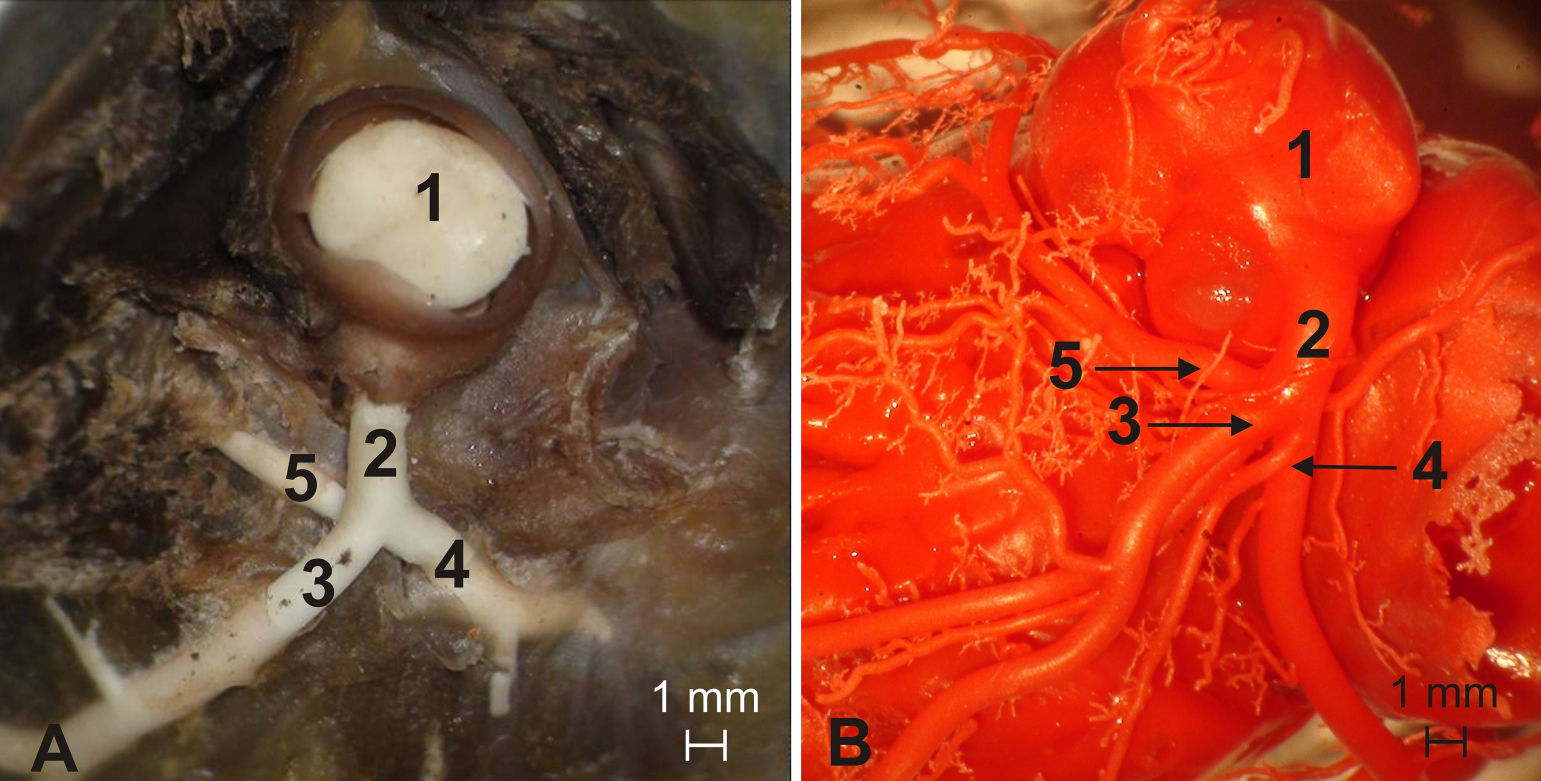
Type III–triple-branched left main stem giving off the left circumflex branch, interventricular branch and the septal branch. 1 –ascending aorta, 2 –left coronary artery (main stem), 3 –interventricular paraconal branch, 4 –left circumflex branch, 5 –septal branch.

Type III (11 animals, 23%)–triple-branched left main stem giving off the left circumflex branch, interventricular branch and the septal branch (Fig 5)

**Fig 6:**
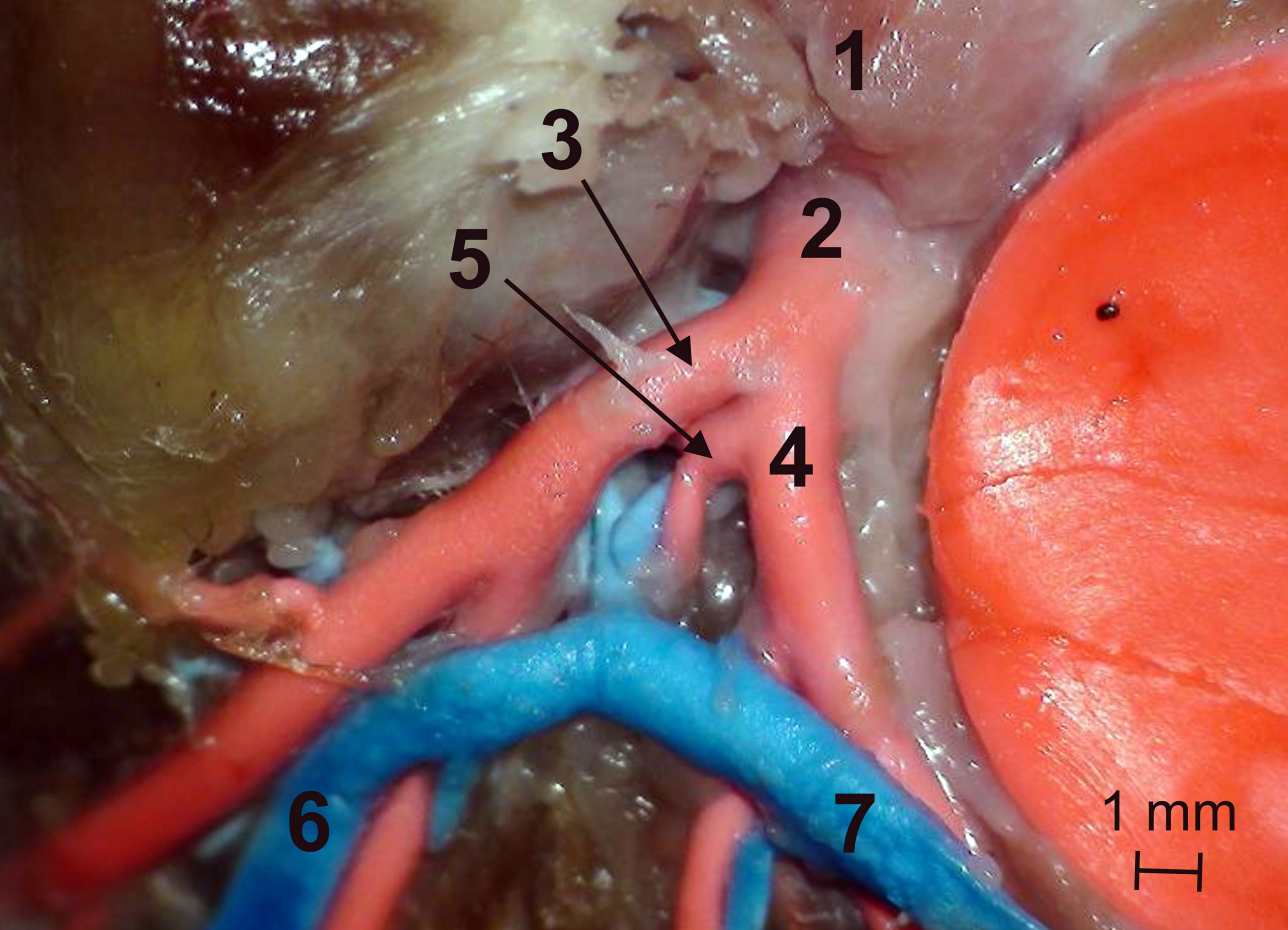
Type IV–double-branched left main stem giving off the interventricular paraconal branch and the left circumflex branch, which gave off the septal branch. 1 –ascending aorta, 2 –left coronary artery (main stem), 3 –interventricular paraconal branch, 4 –left circumflex branch, 5 –septal branch, 6 –great cardiac vein (interventricular paraconal branch), 7 –great cardiac vein (circumflex branch).

Type IV (1 animal, 2%)–double-branched left main stem giving off the interventricular paraconal branch and the left circumflex branch, which gave off the septal branch (Fig 6)

**Fig 7:**
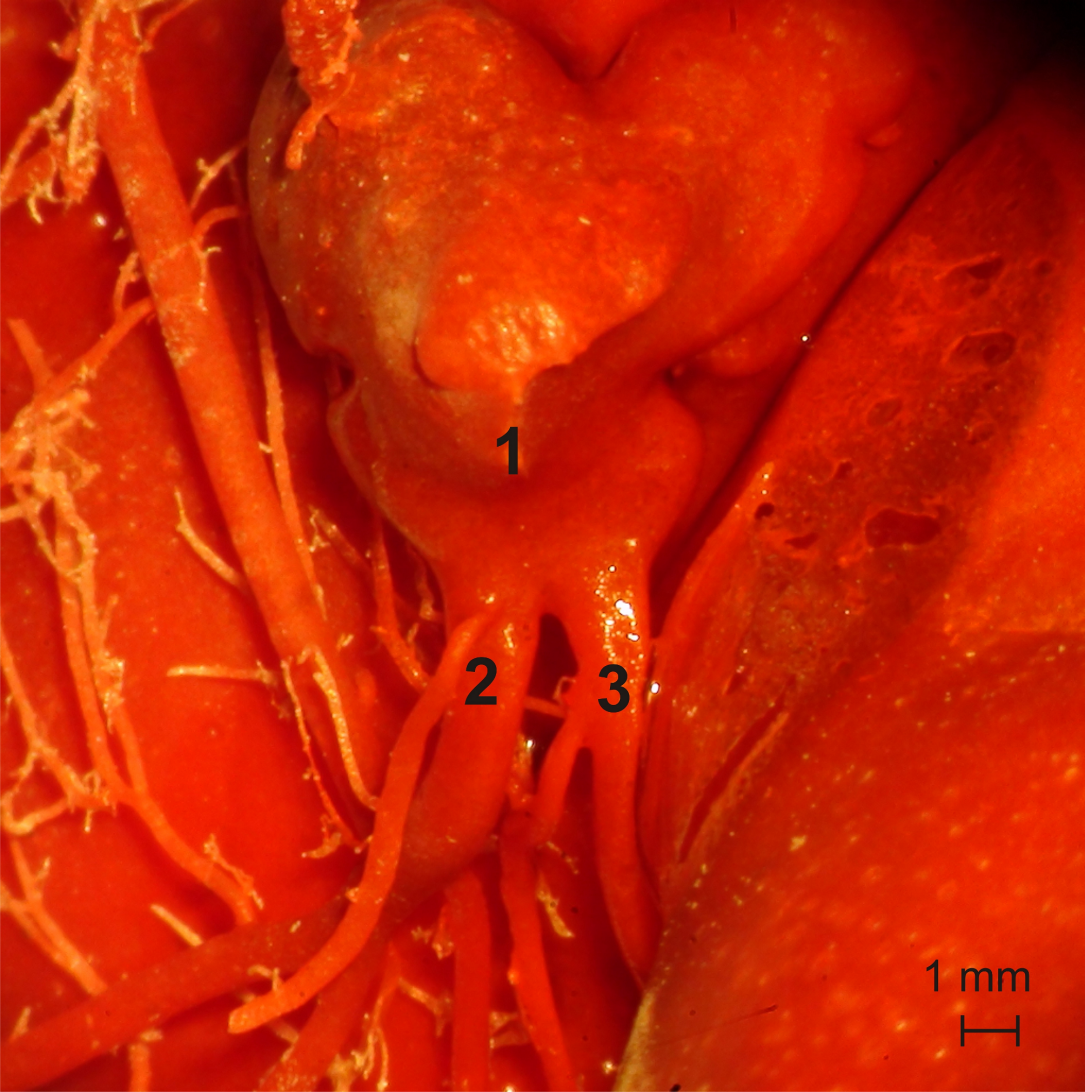
Two separate ostia for the interventricular paraconal branch and the left circumflex branch. 1 –left semilunar leaflet, 2 –interventricular paraconal branch, 3 –left circumflex branch.

One (2%) out of the 48 studied cats had two separate ostia for the interventricular paraconal branch and the left circumflex branch (Fig 7).

A single right stem coronary artery was identified in all the animals. Its coronary ostium was located in the corresponding coronary sinus of the aortic valve. At the base of the heart, it ran between the right auricle and the pulmonary trunk. Then, it entered the coronary sulcus, and reached the atrial surface of the heart as the right circumflex branch.

## Discussion

There are few morphometric studies of the aortic valve and the topography and morphometry of the coronary ostia in veterinary medicine. To date, studies have been carried out in the cow, chicken and donkey [24–26] as well as in monkeys, which are considered an experimental model of the vascular supply of the human heart [12, 15, 27]. Studies in humans present detailed morphometric analyses of individual leaflets of the aortic valve, the diameters of the coronary ostia, their distance from the aortic valve commissures, and the nadirs of the coronary sinuses. These data are used prior to open and endovascular procedures on the aortic valve and coronary arteries [28–35]. In veterinary medicine, such surgical interventions have not been undertaken. However, research in this field as well as increased awareness of pet owners may warrant such procedures in veterinary medicine.

The feline heart is supplied by the left and right coronary artery [2, 16, 20, 36]. We found that the area of the left coronary ostium was larger than the area of the right coronary ostium in all the studied animals. However, the statistical analysis did not reveal gender-dependent differences.

The study carried out by Teofilovski-Parapid et al. [27] in the crab-eating macaque revealed that the diameter of the left coronary artery was 1.2–2.5 mm (on average: 1.8 mm), while the diameter of the right coronary artery was 0.7–1.2 mm (on average: 0.9 mm). The same study also described the morphology of the left main stem coronary artery. The authors found that it was a double-branched artery in 82% of the cases and distinguished the anterior interventricular branch and the circumflex branch. In the remaining 18% of cases, the left main stem was triple-branched. The authors also found a left marginal branch [27]. Studies carried out by Nikolić et al. [12] on the green monkey and crab-eating macaque revealed that the mean diameter of the left coronary artery was 1.65±0.39 mm, and the mean diameter of the right coronary artery was 0.94±0.15 mm. The authors also identified the presence of a third coronary artery (TCA) in 1.8% of the studied monkeys. Buss et al. [37] studied the blood supply to the heart in bonnet monkeys (*Macaca radiata*). However, the findings were limited only to the description of the presence of a double-branched left main stem dividing into the left anterior descending artery and the circumflex artery.

The study by Ozgel et al. [24] on donkeys also revealed that the left coronary artery had a larger diameter (0.9–1 mm) than the right coronary artery (0.1–0.3 mm).

In humans, similar morphometric measurements of the coronary artery ostia were carried out. Kaur et al. [32] reported that of the 77 studied hearts, the diameter of the coronary artery ostia was the same in seven cases, and that the diameter of the right coronary artery ostium was greater than that of the left in 22 cases. Sirikonda and Sreelatha [33] emphasized that in the majority of the hearts they studied (n = 100), the left coronary ostium was larger (4.11±0.88 mm) than the right coronary artery ostium (2.77±0.905 mm). In most studies, only the mean diameters of the coronary ostia were presented. In the study by Bhimalli et al. [31], the mean diameter of the left coronary artery was 3.17±0.34 mm, and the diameter of the right coronary artery was 2.38±1.33 mm. In the study by Cavalcanti et al. [28], those values were 4.25±0.94 mm and 3.46±0.93mm, respectively.

In our study, 36 (75%) of the cats had a double-branched left main stem coronary artery. It divided into the interventricular paraconal branch and the left circumflex branch. Our findings are consistent with those of Vladova [36], who performed a study on 11 cats. However, in that study, no morphological types of the left main stem coronary artery are mentioned. Of the 48 studied cats, 11 (23%) had a triple-branched left main stem coronary artery. Similar studies were carried out in dogs but are inconclusive. Some authors suggest that the majority of dogs have a double-branched left main stem coronary artery (60–80% of the studied animals) [7–9, 38]. According to Blair [7], a triple-branched left main stem coronary artery is the most common form in dogs. In our study group, there were differences in terms of the origin of the septal branch of the left coronary artery were observed. The artery usually originated from the interventricular paraconal branch although in one case it originated in the left circumflex branch. Similar findings were observed in dogs [8, 9, 38]. The absence of the septal branch was noted in 13 cats (27%) from our study group. Similar observations have not been made in dogs.

One cat (2%) from our study group had two separate coronary ostia for the interventricular paraconal branch and the left circumflex branch. Our findings are consistent with the results of an earlier study on the morphology of the coronary artery ostia in the domestic cat [16], where separate ostia were observed in two out of 65 cats. Separate ostia for the interventricular paraconal branch and the left circumflex branch were described in one out of 20 dogs in a study by Noestelthaller et al. [8]. Büll and Martins [38] described such an anatomical variation in 4 (13%) out of 30 dogs.

A double-branched left main stem coronary artery was also noted in the porcupine, ringed seal and Bactrian camel [3–5].

In the Angora rabbit, the left coronary artery was reported to divide into the proximal branch of the left atrium, interventricular paraconal branch and left circumflex branch. Two out of eight rabbits had an additional interventricular septal branch [39].

Numerous studies present descriptions and figures of the blood supply to the heart in different species of domestic animals. However, there are few articles related to the blood vessels of the heart in the cat [16, 36, 40]. Moreover, there have been no studies describing the morphometry of the coronary artery ostia and the anatomy of the proximal segments of the coronary arteries.

New diagnostic and therapeutic methods, commonly used in humans, are introduced in veterinary medicine. These include endovascular procedures, i.e. coronary angiography and angioplasty. Currently, severe and complex feline cardiac diseases are treated.

## Conclusion

The left coronary artery ostium is greater than right. There is considerable diversity in the branches of proximal segment of the left coronary artery, while the right coronary artery is more conservative. The results of this study may aid in defining the normal values for coronary vessels, essential in planning the treatment of heart diseases using endovascular methods.

## Supporting information

S1 Table P_ACS_−the area of the left coronary ostium (mm^2^), P_ACD_−the area of the right coronary ostium (mm^2^), r–difference between P_ACS_ and P_ACD_ (mm^2^). (PDF)

## Abbreviations

KOH: potassium hydroxide
NaOH: sodium hydroxide
P_ACS_: area of the left coronary ostium
P_ACD_: area of the right coronary ostium
r: difference between P_ACS_ and P_ACD_
SCA: single coronary artery
TCA: third coronary artery
