# Supplementary material for: Morphometry of the coronary ostia and the structure of coronary arteries in the shorthair domestic cat
Source: PLoS One. 2017 Oct 11;12(10):e0186177. doi: 10.1371/journal.pone.0186177 (PMC5636138; doi:10.1371/journal.pone.0186177)
Supplement: S1 Table — (PDF) [file pone.0186177.s001.pdf]

nr	sex	PACS	PACD	r
1	♂	0,901	0,314	0,587
2	♂	0,774	0,141	0,633
3	♂	1,794	1,161	0,633
4	♂	1,028	0,313	0,715
5	♂	1,569	0,381	1,188
6	♂	1,08	0,282	0,798
7	♂	0,799	0,719	0,08
8	♂	0,89	0,582	0,308
9	♂	1,362	0,917	0,445
10	♂	1,223	0,882	0,341
11	♂	1,224	0,32	0,904
12	♂	1,409	0,625	0,784
13	♂	0,827	0,126	0,701
14	♂	2,644	1,303	1,341
15	♂	1,34	1,104	0,236
16	♂	1,497	0,315	1,182
17	♂	1,635	0,544	1,091
18	♂	0,712	0,636	0,076
19	♂	1,199	0,277	0,922
20	♂	0,658	0,472	0,186
21	♂	0,535	0,118	0,417
22	♂	0,825	0,749	0,076
23	♂	1,105	0,244	0,861
24	♂	0,856	0,219	0,637
25	♂	0,919	0,324	0,595
26	♂	1,015	0,154	0,861
27	♀	1,195	0,254	0,941
28	♀	0,713	0,558	0,155
29	♀	1,347	0,38	0,967
30	♀	1,135	0,599	0,536
31	♀	1,581	0,233	1,348
32	♀	0,774	0,21	0,564
33	♀	1,085	0,294	0,791
34	♀	0,638	0,628	0,01
35	♀	1,55	1,37	0,18
36	♀	0,918	0,329	0,589
37	♀	2,495	0,795	1,7
38	♀	0,563	0,184	0,379
39	♀	0,664	0,403	0,261
40	♀	0,821	0,312	0,509
41	♀	0,774	0,428	0,346
42	♀	1,073	0,225	0,848
43	♀	0,95	0,197	0,753
44	♀	1,109	0,441	0,668
45	♀	1,092	0,288	0,804
46	♀	1,353	0,999	0,354
47	♀	1,526	0,63	0,896
48	♀	1,766	0,321	1,445
49	♀	1,212	0,37	0,842
50	♀	1,001	0,701	0,3
51	♀	0,786	0,213	0,573
52	♀	1,192	0,838	0,354
